# Exploring the Link Between Traumatic Brain Injury and Benign Paroxysmal Positional Vertigo

**DOI:** 10.7759/cureus.81847

**Published:** 2025-04-07

**Authors:** Melissa Castillo-Bustamante, Bernardo F Ramos, Susan Whitney, Francisco Zuma e Maia, Renato Cal, Jorge Madrigal

**Affiliations:** 1 Otolaryngology, Clinica Universitaria Bolivariana, Medellín, COL; 2 College of Medicine, Health Sciences School, Universidad Pontificia Bolivariana, Medellín, COL; 3 Otolaryngology, Federal University of Espirito Santo, Vitoria, BRA; 4 Physical Medicine and Rehabilitation, University of Pittsburgh, Pittsburgh, USA; 5 Otolaryngology, Clinica Maia, Canoas, BRA; 6 Otolaryngology, University Center of the State of Pará (CESUPA), Belem, BRA; 7 Otoneurology, Centro de Vértigo y Mareo, Mexico City, MEX

**Keywords:** benign paroxysmal positional vertigo (bppv), otolithic organs, post-traumatic vertigo, traumatic brain injury (tbi), vestibular disorders, vestibular rehabilitation

## Abstract

Benign paroxysmal positional vertigo (BPPV) is the most common peripheral vestibular disorder, characterized by brief episodes of vertigo triggered by changes in head position. While idiopathic cases are frequent, post-traumatic BPPV has been increasingly recognized, particularly in individuals who have experienced traumatic brain injury (TBI). TBI, ranging from mild concussions to severe head trauma, is a significant cause of neurological morbidity and is often associated with vestibular dysfunction. The pathophysiology of post-traumatic BPPV is thought to involve direct mechanical disruption of the otolithic organs, alterations in endolymph dynamics, or vascular compromise affecting inner ear structures. Compared to idiopathic BPPV, post-traumatic cases tend to have a more prolonged and refractory course, often requiring multiple repositioning maneuvers for symptom resolution.

Additionally, concurrent vestibular pathologies, such as vestibular migraine, post-concussive dizziness, or central vestibular dysfunction, may complicate diagnosis and treatment. Early identification and appropriate management of post-traumatic BPPV are crucial in reducing disability and improving the quality of life in affected patients. This review explores the epidemiology, pathophysiology, clinical characteristics, and treatment considerations of post-traumatic BPPV, emphasizing the importance of a multidisciplinary approach. Understanding the relationship between TBI and BPPV can enhance clinical decision-making and optimize rehabilitation strategies for individuals with vestibular dysfunction following head trauma.

## Introduction and background

Benign paroxysmal positional vertigo (BPPV) is a highly prevalent and well-recognized condition within the spectrum of vestibular disorders, often manifesting as transient episodes of vertigo provoked by specific changes in head position [1]. These episodes are typically short-lived but can be profoundly disorienting and disruptive to daily life, leading to significant physical and emotional distress [1]. While BPPV frequently occurs as an idiopathic condition, its association with traumatic brain injury (TBI) has become an area of growing clinical and research interest [2]. TBI encompasses a wide range of injuries, from mild concussions to severe, life-threatening trauma, and remains a leading contributor to global neurological morbidity and long-term disability [3]. The prevalence of vestibular dysfunction following TBI underscores the importance of understanding and addressing these complications, with BPPV representing a particularly impactful sequela [4]. Vestibular disorders such as BPPV can profoundly impair a patient’s quality of life, limiting functional independence and impeding recovery from the primary brain injury [4].

The interplay between TBI and BPPV is complex and multifactorial, involving distinct yet overlapping mechanisms that challenge clinicians in both diagnosis and management [4]. This narrative review seeks to provide a comprehensive examination of the relationship between these conditions, delving into the underlying pathophysiology, epidemiological patterns, and unique clinical challenges associated with BPPV in the context of TBI.

## Review

Methods

This narrative review synthesizes findings from a comprehensive literature search conducted across multiple electronic databases, including PubMed, Scopus, and Web of Science. Relevant articles published from 2000 to 2024 were identified using search terms such as "benign paroxysmal positional vertigo," "BPPV," "traumatic brain injury," "TBI," "vestibular dysfunction," "vestibular rehabilitation," and related keywords. Both peer-reviewed original research articles and review papers were included to ensure a broad perspective on the topic. Studies focusing on the pathophysiology, epidemiology, diagnostic challenges, treatment strategies, and rehabilitation outcomes of BPPV in the context of TBI were prioritized.

The inclusion criteria for this review were studies that explicitly addressed BPPV secondary to TBI or included data stratifying idiopathic and post-traumatic cases, articles published in English, and articles reporting primary data or critical analyses relevant to clinical practice or research in this field. Exclusion criteria encompassed studies with insufficient detail on methodology, those limited to case reports without broader applicability, and articles focusing exclusively on other vestibular disorders without specific reference to BPPV. Data extraction and synthesis were performed iteratively, focusing on key themes such as the mechanisms of otoconia displacement in TBI, epidemiological distinctions between idiopathic and post-traumatic BPPV, and the efficacy of diagnostic and therapeutic approaches. Controversies, limitations, and gaps in the literature were also critically examined. The findings were then organized into thematic sections to provide a coherent narrative aligned with the objectives of this review.

Pathophysiology

The pathophysiology of BPPV centers on the displacement of otoconia, which are calcium carbonate crystals located on the utricular macula within the vestibular system [5]. In BPPV, these otoconia become dislodged and migrate into the semicircular canals, most commonly the posterior canal [6]. Once in the canals, the otoconia act as “foreign bodies,” disrupting the normal flow of endolymphatic fluid during head movements [7]. This disruption leads to abnormal deflection of the ampullary cupula and overstimulation of the vestibular nerve, manifesting as the characteristic episodic vertigo triggered by positional changes [7]. While this mechanism is well-documented in idiopathic BPPV, its manifestation following traumatic brain injury (TBI) involves additional complexities [2].

In the context of TBI, the displacement of otoconia is attributed to a combination of mechanical, ischemic, and metabolic factors [2,8]. Direct mechanical trauma to the inner ear during a TBI can result in significant shearing forces, causing physical detachment of otoconia from the utricular macula [2,8]. This is especially pronounced in cases involving temporal bone fractures, where the structural integrity of the labyrinth is directly compromised [2,8]. Even in the absence of fractures, rapid deceleration or rotational forces during TBI can impose sufficient stress on the otolithic organs to induce otoconia displacement [9]. The pathophysiological changes extend beyond mechanical effects, with TBI-induced ischemia and inflammation in the vestibular apparatus playing a crucial role [10,11]. These processes can weaken the adhesive glycoproteins that secure the otoconia to the utricular macula surface, increasing their susceptibility to detachment [11,12].

Emerging evidence suggests that secondary biochemical alterations following TBI further exacerbate the risk of BPPV [13]. Changes in calcium metabolism, often linked to systemic and local inflammatory responses, can destabilize the homeostasis of the vestibular end organs [13]. Oxidative stress, another consequence of TBI, has been implicated in cellular damage within the vestibular system, potentially compromising the resilience of the otoliths and their anchoring structures [14]. Notably, the etiology of BPPV differs depending on whether the TBI involves a fracture. In cases of temporal bone fractures, the direct disruption of the membranous labyrinth is a primary contributor [15]. Conversely, in TBI without fractures, indirect mechanisms such as intracranial pressure changes, ischemic injury, and metabolic dysregulation predominate [14,15].

Despite these insights, the pathophysiology of post-traumatic BPPV remains incompletely understood [15]. The variability in clinical presentation, ranging from isolated vertigo to complex vestibular and balance disturbances, suggests the involvement of additional, as-yet-unidentified factors [16]. These could include genetic predispositions, variability in vestibular anatomy, or differences in the inflammatory and reparative responses following TBI [16]. Further research is needed to elucidate these mechanisms and their implications for personalized management strategies in patients with TBI-associated BPPV [16].

Epidemiology

BPPV is a well-documented sequela of TBI, with studies estimating that up to 28% of patients with head trauma develop this condition [17]. This prevalence is notably higher than that observed in the general population, underscoring the unique vulnerability of individuals with TBI to developing vestibular dysfunction [17]. Post-traumatic BPPV, however, displays a broader age distribution and is often observed in younger patients, reflecting the demographic profile of persons post-TBI, which frequently includes adolescents and young adults involved in high-impact activities such as sports or motor vehicle accidents [16].

The latency period between the traumatic event and the onset of BPPV symptoms varies significantly, ranging from days to several months [18]. This variability complicates the recognition of a causal relationship, particularly when symptoms emerge long after the acute phase of TBI management [18]. Such delays may result from gradual biochemical and structural changes within the vestibular apparatus or from secondary factors such as delayed otoconia detachment due to cumulative stress on weakened otolithic membranes [18].

Epidemiological studies also reveal that post-traumatic BPPV is more likely to involve bilateral presentation and atypical canal involvement, including the lateral and anterior semicircular canals [15,19]. The higher prevalence of bilateral and atypical cases in post-traumatic BPPV may be attributed to the diffuse nature of traumatic forces acting on the head and inner ear, leading to widespread disruption of vestibular structures [15,19]. These patterns emphasize the need for heightened clinical vigilance in diagnosing BPPV in patients with a history of TBI, as standard diagnostic maneuvers may be insufficient to detect atypical presentations [15,19].

Moreover, the potential for coexisting vestibular disorders or central nervous system involvement in TBI patients complicates the clinical picture further [4]. Differentiating post-traumatic BPPV from other vestibular conditions such as vestibular migraine, persistent postural-perceptual dizziness, or central vestibular dysfunction requires a comprehensive and nuanced approach [20].

Risk factors

The development of vertigo in individuals with traumatic brain injury (TBI), whether with or without fractures, is influenced by a variety of risk factors that can complicate the clinical course and impact recovery [4]. For TBI patients, the risk of developing vertigo is increased by the nature and severity of the injury, as well as additional coexisting factors [4]. Some of the following may be considered risk factors.

Severity of TBI

The severity of the brain injury is a critical risk factor for developing vertigo [2]. Severe TBI, particularly those involving diffuse axonal injury or significant brain contusions, is more likely to result in vestibular dysfunction [21]. These injuries may disrupt the central vestibular pathways, leading to vertigo symptoms [21]. Mild TBIs (concussions) can also result in vertigo, though the mechanisms are less clear, and symptoms may be transient or less severe [22].

Fractures Involving the Skull or Cervical Spine

Patients with fractures, particularly those affecting the skull or cervical spine, are at higher risk of developing vertigo [23]. Skull fractures may directly damage the inner ear structures or disrupt the vestibular system, while cervical spine fractures can lead to proprioceptive deficits and neck-related vertigo [23]. These fractures can limit the rehabilitation process and complicate the management of vertigo [23].

Inner Ear Injury or Damage

Direct injury to the inner ear structures, including the cochlea or vestibular apparatus, is another significant risk factor [21]. Fractures or trauma to the temporal bone can cause damage to the otolithic organs or semicircular canals, which are involved in balance control [21]. These injuries can result in BPPV or other vestibular disorders [22,23].

Prolonged Immobilization

Prolonged lying in one position after TBI can worsen vestibular dysfunction by limiting sensory input, increasing dizziness upon movement, and delaying recovery of balance and spatial orientation systems [23].

Age and Pre-existing Health Conditions

Older adults with TBI are more likely to experience long-term vestibular symptoms, including vertigo [12]. Age-related declines in the vestibular system and musculoskeletal health can make recovery more challenging [12]. Pre-existing conditions such as diabetes, hypertension, cardiovascular diseases, osteoporosis, and osteopenia may also increase the risk of complications and worsen balance issues following TBI [12].

Psychological Factors

Anxiety, depression, and post-traumatic stress disorder (PTSD) are common among TBI patients and can heighten sensitivity to vestibular symptoms [24]. These psychological factors can amplify the perception of vertigo and affect the overall ability to cope with the condition, leading to a worse quality of life [24].

Delayed Rehabilitation or Inadequate Treatment

Delayed initiation of rehabilitation or inadequate treatment may also increase the risk of persistent vertigo in TBI patients [25]. Early intervention is critical to capitalize on neuroplasticity and prevent the worsening of vestibular dysfunction [25]. Without proper rehabilitation, TBI patients, especially those with fractures, may experience prolonged symptoms and difficulties in achieving functional recovery [25]. Traumatic BPPV appears to be more challenging to treat than non-traumatic causes of BPPV and recurrence is more frequent [26-28].

Clinical Challenges and Diagnostic Considerations

Diagnosing BPPV in patients with TBI presents a range of unique and significant challenges. The overlap of symptoms commonly associated with TBI, including dizziness, imbalance, headache, cognitive dysfunction, and fatigue, often obscures the clinical picture and makes it difficult to isolate BPPV as the primary cause of a patient’s vertigo [2]. The subjective nature of dizziness-related complaints in these patients further complicates the diagnostic process, as symptoms vary widely in intensity, duration, and triggers [2]. In many cases, patients may have difficulty articulating or associating their symptoms with specific positional changes, leading to delays in diagnosis or misdiagnosis [2,18].

Clinical assessment in patients with TBI has been documented in the literature to include ophthalmoscopy, otoscopy, eye movement evaluation (cover test, gaze testing, saccades, smooth pursuit in the videonystagmography {VNG}, and vestibulo-ocular reflex {VOR} via the video head impulse test {vHIT}), the Dix-Hallpike maneuver, and gait assessment (Romberg test for 20 seconds, tandem walking, and tandem stance). When possible, clinical findings are confirmed through laboratory testing, such as otolith testing (including cervical and ocular vestibular-evoked myogenic potentials (c and o VEMPs) [4,12,22,25,26].

Traditional diagnostic maneuvers, such as the Dix-Hallpike test and head-roll test, remain the gold standard for identifying BPPV [29]. However, their utility in the TBI population can be limited due to various factors [29]. For instance, patients with cervical spine injuries or chronic neck pain, common comorbidities in TBI, may be unable to tolerate the required head and neck movements [30]. Moreover, the presence of vestibular hypofunction secondary to TBI can diminish the intensity of nystagmus responses during diagnostic testing, potentially leading to false-negative results [4]. Given these diagnostic complexities, a multidisciplinary approach is often necessary to ensure a comprehensive assessment [12]. Collaboration among otolaryngologists, neurologists, physiotherapists, and rehabilitation specialists can help address the multifaceted needs of TBI patients with suspected BPPV [12]. Such an approach not only enhances diagnostic accuracy but also facilitates the development of personalized management strategies tailored to the unique challenges faced by this patient population [3]. Furthermore, incorporating psychological support into the diagnostic and treatment process is essential, as many TBI patients experience anxiety or depression that can exacerbate dizziness and complicate symptom interpretation [3].

VNG findings have shown that patients tested positive for BPPV during vestibular and balance assessments conducted months after TBI, all presenting nystagmus profiles consistent with posterior canalithiasis [4,12]. Among these, some experienced recurrent positional vertigo within a short time after injury, with variable responses to previous repositioning maneuvers. Additionally, several patients who tested negative at the time of assessment had a documented history of BPPV treatment since their injury [4,12]. Abnormalities in central oculomotor function were identified in a significant number of cases with available data [4,12]. The most common finding was abnormal smooth pursuit with low gain, followed by prolonged saccade latencies, reduced saccade velocities, bidirectional gaze-evoked or vertical nystagmus in darkness, poor VOR suppression, and inaccurate saccades [4,12]. VNG findings have also documented that a significant proportion of patients with interpretable results exhibited low-velocity nystagmus, which was horizontal and unidirectional in one or more gaze positions in darkness [4,12]. In cases of vestibular hypofunction, both ipsilesional and contralesional quick phases were observed [4,12].

In patients with TBI, the horizontal semicircular canal was the most frequently affected, as identified through a combination of vHIT and caloric testing [4,12,22]. However, caloric testing demonstrated a slightly higher detection rate compared to vHIT, with some cases exhibiting dissociated results (caloric hyporeflexia with a normal vHIT) [4,12,22]. These discrepancies suggest that while vHIT is valuable for detecting high-frequency vestibular deficits, it may not fully capture dysfunction identified by caloric testing, which assesses the vestibulo-ocular reflex at lower frequencies [4,12,22]. Additionally, abnormalities were observed in the posterior and anterior semicircular canals. In several cases, vestibular deficits were unilateral, reinforcing the need for comprehensive vestibular assessment in TBI patients [4,12,22].

Post-urography findings have highlighted notable balance deficits in patients with TBI [12]. A significant number of cases showed loss of balance classified as a “fall” by the software, particularly in conditions requiring high vestibular input [12]. Some patients also exhibited falls in conditions assessing somatosensory and visual contributions to balance. Most patients had abnormal results in one or more sensory organization test (SOT) scores [12]. Deficits were most frequently observed in tasks requiring vestibular, visual, and somatosensory input, with a substantial proportion demonstrating impairments across multiple sensory domains [12]. Additionally, some patients showed difficulty ignoring incorrect visual information, suggesting a possible over-reliance on vision for balance [12].

Otolith function assessment using VEMPs has shown varying rates of saccular dysfunction, with cervical vestibular evoked myogenic potentials (cVEMPs) frequently used to evaluate TBI-related vestibular impairment [12]. Utricular dysfunction assessment through ocular vestibular evoked myogenic potential (oVEMPs) has been less commonly reported, with studies showing mixed findings regarding its prevalence in TBI patients [12]. Some studies have identified asymmetrical oVEMP responses, while others have reported a higher incidence of abnormalities in patients with post-traumatic episodic vertigo [12].

Abnormalities were observed across different vestibular structures, including the horizontal semicircular canal, saccule, utricle, and posterior and anterior canals [12]. While the overall frequency of abnormalities was not high, findings suggest that TBI can potentially affect any part of the labyrinth or vestibular nerve [12]. Some studies have proposed that TBI predominantly impacts the inferior vestibular nerve, with combined posterior canal and saccular dysfunction observed in several cases [12]. Others suggest a greater likelihood of otolith involvement, based on high cVEMP and subjective visual vertical abnormalities recorded in the acute and sub-acute phases [12]. Both patterns of dysfunction were observed, but neither was consistent enough to be considered a hallmark of chronic TBI [12].

Different patterns of vestibular end-organ and nerve dysfunction may be associated with different types of head injuries [4,12,22]. Occipital impacts are more likely to cause skull fractures that breach the otic capsule, leading to vestibular dysfunction and other intracranial complications [4,12,22]. Injuries involving angular head acceleration may make the semicircular canals more susceptible to damage, whereas blast-related injuries involve shock waves that propagate through fluid spaces, including the inner ear via the CSF and ossicular chain [4,12,22]. Given their proximity to the stapes footplate and cochlear oval window, the otolith organs may be particularly vulnerable in such cases [4,12,22]. This could explain the high rate of abnormal cVEMP responses reported in blast-injured patients and suggests that this type of trauma should be considered separately when evaluating the vestibular effects of TBI [4,12,22,25]. Additionally, lateral skull impacts, especially those involving implosive forces through the external auditory canal, may similarly place the otolith organs at risk [4,12,22,25].

The Thomas Richard Vitton (TRV) repositioning chair addresses the challenges of managing BPPV in patients with TBI and cervical spine injuries by enabling precise, controlled diagnostic and therapeutic maneuvers while minimizing strain on the neck [31]. Its design allows for the safe execution of tests like the Dix-Hallpike and head-roll tests, even in patients unable to tolerate traditional techniques [31]. Additionally, the chair ensures accurate otolith repositioning and supports vestibular rehabilitation, addressing the complex interplay of vestibular dysfunction and motor control deficits in TBI [31]. This technology highlights the importance of patient-centered, multidisciplinary care, improving outcomes in this challenging population [32].

In acute TBI, vestibular dysfunction may be under-recognized, as half of the patients with clear gait ataxia reported no balance problems [4]. This aligns with previous findings where no correlation was found between objective imbalance signs and self-reported symptoms [4]. While protein biomarkers like S100B show high sensitivity for mild TBI (mTBI), their low specificity limits their diagnostic utility, especially when imaging and neurological exams appear normal [4]. The spectrum of mTBI ranges from no detectable brain injury to microstructural changes and diffuse axonal injury (DAI), the latter being the most severe and associated with disruption of white matter tracts [4]. DAI occurs due to axonal traction forces affecting the cytoskeleton and impairing axoplasmic transport, typically involving the gray-white junctions, corpus callosum, brainstem, and cerebellar peduncles [4]. While conventional MRI may not detect all structural changes, advanced techniques like diffusion tensor imaging (DTI) can reveal disruptions in white matter integrity [4]. Experimental models of mTBI continue to explore its underlying mechanisms, highlighting the complex interplay of axonal and white matter injury in post-concussion syndrome [4].

Controversies and Facts

Several controversies surround the management of benign paroxysmal positional vertigo (BPPV) in the context of traumatic brain injury (TBI) [31]. One of the key challenges is the timing of vestibular rehabilitation [32]. While early intervention is often recommended to leverage the brain’s neuroplasticity and accelerate functional recovery, there are concerns about the potential for premature therapy to exacerbate symptoms such as dizziness or trigger secondary injuries [2,3]. This is particularly concerning in patients with severe TBI or additional musculoskeletal injuries, where the risk of overloading the system is heightened [2,32]. Balancing the benefits of early rehabilitation against the potential for harm remains a difficult issue in clinical practice [2,32]. Since the presence of BPPV has been related to falls, early rehab might decrease the chance of a fall early in their TBI recovery [33-37].

Another area of debate is the efficacy of canalith repositioning maneuvers (CRMs) in post-traumatic BPPV [36]. While these maneuvers, such as the Epley and Semont techniques, are well-established and highly effective for idiopathic BPPV, their success is less consistent in TBI patients [3]. Some clinicians advocate for modified or repeated maneuvers to address these challenges, while others recommend combining CRMs with vestibular rehabilitation exercises to improve treatment outcomes [20]. The Li maneuver is a promising intervention as it is like the Semont maneuver, yet no head rotation is required for posterior canal BPPV [36]. The Li quick repositioning maneuver is ideal for treating persons with lateral canal BPPV, as they only need to be rolled from one side to the other for an effective intervention [36].

In TBI patients, particularly those with fractures, there are added complications in the rehabilitation process [37]. Fractures, especially in the weight-bearing joints or spine, can restrict movement and hinder the implementation of certain balance training exercises [34]. The pain associated with these fractures often limits participation in therapy, while prolonged immobilization can exacerbate vestibular symptoms and lead to secondary issues such as muscle weakness and joint stiffness [3]. For patients without fractures, although the rehabilitation approach may be less constrained physically, cognitive impairments and anxiety, which are common in TBI patients, can still complicate the effectiveness of BPPV treatment [28]. Early rehab after moderate-to-severe brain injury results in better outcomes [38].

The recurrence rate of BPPV in TBI patients is another contentious issue [38]. Some evidence suggests that BPPV may recur more frequently in this population compared to idiopathic cases, potentially due to persistent structural vulnerabilities or difficulties in otoconial regeneration [39]. This issue is especially pronounced in patients with fractures, where the risk of vestibular dysfunction may be higher [39]. Pharmacological interventions, such as calcium-channel stabilizers or supplements aimed at improving inner ear metabolism, have been proposed to reduce recurrence rates [39]. However, the lack of large-scale studies leaves clinicians uncertain about the efficacy of these treatments, underscoring the need for more robust research [39].

Psychosocial challenges in managing BPPV in TBI patients cannot be overlooked [40]. Anxiety, depression, agitation, and post-traumatic stress disorder are prevalent in this group and can worsen dizziness symptoms or impede progress in rehabilitation [18]. The debate continues regarding the extent to which psychological support should be integrated into BPPV treatment [40-42]. Some advocate for a multidisciplinary approach that includes mental healthcare, while others suggest that symptoms may resolve through physical therapy alone, without the need for extensive psychological intervention [42].

The impact of traumatic brain injury (TBI) with and without fractures on the quality of life (QoL) of individuals experiencing vertigo is significant and multifaceted [3]. For TBI patients, vertigo is a debilitating symptom that can severely affect daily functioning, emotional well-being, and overall quality of life [3]. In patients with TBI without fractures, the presence of vertigo in these individuals can lead to heightened anxiety, depression, and increased risk of falls, which, in turn, can exacerbate their functional limitations and further decrease their independence [42]. Cognitive difficulties, such as memory and attention deficits, can compound the challenges of managing vertigo, creating a cycle of frustration and worsening symptoms that directly affect the quality of life [42].

Not all individuals with TBI and BPPV report experiencing spinning or dizziness, which can make the diagnosis of BPPV challenging [18]. They may report that they have nausea or that they feel lightheaded, but do not report vertigo [40]. One must investigate the possibility of BPPV in persons after a brain injury or anyone who has fallen [42,43].

**Figure 1:**
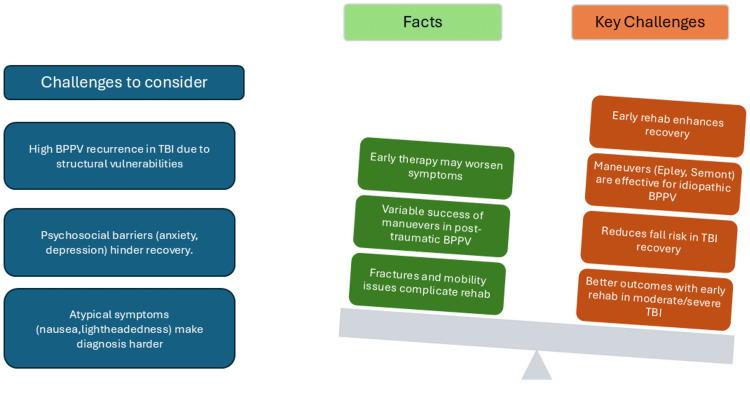
Challenges to consider, facts, and key challenges in traumatic brain injury and benign paroxysmal positional vertigo. TBI: traumatic brain injury; rehab: rehabilitation; BPPV: benign paroxysmal positional vertigo The image is created by the authors of this study.

The key challenges, challenges to consider, and relevant facts related to TBI and vertigo are summarized in Figure 1. Providing further details on these aspects could enhance clarity and offer a more comprehensive understanding of their impact and clinical implications.

Vestibular Rehabilitation and Challenges

Vestibular rehabilitation plays a critical role in the management of benign paroxysmal positional vertigo (BPPV) in traumatic brain injury (TBI) patients, offering a pathway to improve overall balance and quality of life [42-44]. The primary goals of rehabilitation are to restore balance, alleviate vertigo symptoms, and promote functional independence [43-45]. For TBI patients, these goals become more difficult to achieve due to the presence of multiple complicating factors [43-45]. Cognitive deficits, such as memory impairment or difficulties with attention and processing speed, often interfere with the patient’s ability to fully engage in therapy [45]. Additionally, emotional challenges like anxiety and depression, which are common in individuals recovering from TBI, can further hinder progress [44-46]. Impaired proprioception, or the inability to sense body position accurately, is also a frequent issue in TBI patients and can limit the effectiveness of traditional vestibular rehabilitation techniques [45]. These challenges necessitate individualized, multidisciplinary rehabilitation plans that can address the unique needs of each patient, making the process not only more complex but also more resource-intensive [46].

Customizing rehabilitation protocols to effectively manage these coexisting issues is essential, including the incorporation of cognitive exercises, psychological support, and adjustments to physical therapy approaches [47]. However, the added time and effort involved can strain healthcare resources and require specialized training for practitioners [47]. Furthermore, the effectiveness of vestibular rehabilitation in TBI-related BPPV has not been as well-documented or studied as its application in idiopathic BPPV cases [46]. While there is a growing body of evidence supporting rehabilitation for idiopathic BPPV, more research is needed to understand how TBI specifically influences the pathophysiology of BPPV, how rehabilitation protocols can be adjusted to address this, and whether TBI patients respond as effectively as those with idiopathic conditions [47].

Emerging therapeutic modalities, such as virtual reality-based balance training, sensorimotor integration therapies, and advanced neuroplasticity-focused interventions, promise to improve vestibular function in TBI patients [48]. Advanced approaches include non-invasive brain stimulation techniques like transcranial magnetic stimulation (TMS) and transcranial direct current stimulation (tDCS), which aim to enhance neural plasticity and facilitate vestibular compensation [48]. Additionally, wearable biofeedback devices, such as inertial sensors and postural sway monitoring systems, provide real-time feedback to patients, promoting better balance control and motor learning [48]. Robotics-assisted rehabilitation, incorporating exoskeletons or balance-training platforms, further enhances dynamic stability and proprioceptive training [48]. These strategies, combined with traditional vestibular rehabilitation, hold great potential for optimizing recovery and functional outcomes in individuals with TBI-related vestibular dysfunction [48]. These methods offer the potential for more engaging and targeted rehabilitation, particularly for patients struggling with cognitive and proprioceptive impairments [48]. However, the evidence supporting these novel interventions is still in its early stages [48]. Larger, more controlled studies are necessary to validate these emerging treatments and establish their efficacy in improving outcomes for TBI-related BPPV [48].

The primary objectives of rehabilitation are to restore balance, reduce vertigo, and enhance functional independence [49]. However, TBI patients often face additional difficulties, especially when factors such as fractures or other neurological impairments are present [49]. For individuals with both TBI and fractures, rehabilitation can be particularly complicated due to the added physical limitations [50]. Fractures can restrict movement and complicate balance exercises, which in turn limit the effectiveness of traditional rehabilitation methods [50]. The pain resulting from fractures may also discourage active participation in therapy, while fractures involving weight-bearing joints can restrict certain exercises, slowing recovery [50]. Furthermore, fractures, particularly those affecting the spine or extremities, may necessitate extended periods of immobilization, which can worsen vestibular symptoms and lead to secondary complications such as muscle weakness or joint stiffness [21].

In contrast, TBI patients without fractures still face significant rehabilitation challenges due to other factors, such as cognitive deficits, impaired proprioception, and psychological barriers like anxiety and depression [51]. Cognitive impairments, such as difficulties with attention, memory, and executive function, can impede the patient's ability to engage in and retain rehabilitation exercises fully [51]. Anxiety or depression, which are common in TBI patients, can further hinder progress by reducing motivation and complicating therapy adherence [51]. Impaired proprioception, often seen in TBI patients, diminishes their awareness of body position, making balance exercises more difficult and less effective [51].

Customizing rehabilitation protocols to address these coexisting challenges is essential, but it requires careful coordination and can be resource-intensive [52]. For TBI patients with fractures, therapy may need to incorporate additional strategies, such as pain management, adaptive movement techniques, and careful monitoring of the healing process to avoid re-injury [52]. For those without fractures, rehabilitation programs must be tailored to address cognitive, psychological, and proprioceptive difficulties, often requiring multidisciplinary interventions [52]. Furthermore, the effectiveness of rehabilitation in TBI-related BPPV remains less well-documented compared to idiopathic cases, particularly when fractures are involved [52]. More research is needed to understand how these complications affect rehabilitation outcomes and to develop evidence-based protocols that cater to these specific needs.

The management of BPPV in traumatic brain injury (TBI) patients, particularly those with fractures, presents a complex set of challenges and limitations. One of the primary difficulties lies in the limited and inconsistent research regarding the effectiveness of current treatment protocols for post-traumatic BPPV, especially when compared to idiopathic cases [2]. Although vestibular rehabilitation and canalith repositioning maneuvers (CRMs) are widely used, their success in TBI patients remains inadequately documented, and the presence of coexisting injuries or cognitive impairments often complicates treatment [2]. Additionally, the recurrence rate of BPPV in TBI patients is a contentious issue, with a lack of large-scale studies to guide clinical practice [3]. The high cost and limited availability of diagnostic tools, such as video-oculography and vestibular evoked myogenic potentials, further constrain optimal care [3].

As part of the treatment for BPPV, the minimal stimulus strategy could prove crucial [52]. This approach involves performing diagnostic and therapeutic maneuvers with the least number of head and body movements necessary to achieve accurate assessment and effective treatment outcomes [53]. This strategy is particularly important for patients with comorbidities such as traumatic brain injury (TBI) or cervical spine injuries, where excessive movement may trigger pain, discomfort, or exacerbate symptoms [53]. By minimizing the intensity and duration of movement, the risk of inducing severe vertigo or nausea is reduced, which improves patient tolerance and adherence to treatment [53]. Additionally, this approach allows for more controlled and precise repositioning of the otoliths during therapeutic maneuvers, such as the Epley or Semont maneuvers, optimizing therapeutic results while minimizing discomfort [53]. Incorporating this strategy into treatment protocols could significantly enhance outcomes and the patient experience, particularly for those with complex conditions [53].

It is also important to emphasize the value of assessing patients with BPPV, particularly those who have experienced traumatic brain injury (TBI), using videonystagmography (VNG) [54]. VNG allows for a more precise evaluation of nystagmus and can help identify subtle vestibular dysfunctions that may not be detectable through clinical observation alone [54]. When available, the use of Frenzel video goggles can further enhance diagnostic accuracy by providing a more controlled visual environment [55]. This enables clinicians to observe and analyze eye movements in real-time without the influence of visual distractions [55]. The combination of these diagnostic tools is crucial in providing a comprehensive evaluation and ensuring appropriate management of BPPV in TBI patients [55].

In this context, the integration of technologies like the TRV repositioning chair has the potential to enhance both diagnostic accuracy and therapeutic outcomes [34]. The TRV chair offers precise control over the head and body during diagnostic and therapeutic maneuvers, allowing clinicians to effectively manage BPPV, even in patients with cervical spine injuries or those unable to tolerate traditional tests [34]. This tool could bridge the gap in treating post-traumatic BPPV by offering a safer, more efficient means of repositioning otoliths and supporting vestibular rehabilitation [34].

Looking toward the future, there is a clear need for more robust, large-scale studies to better understand the mechanisms underlying BPPV in TBI patients and to refine treatment strategies [3]. Emerging therapies, such as virtual reality-based rehabilitation and pharmacological interventions aimed at reducing recurrence, show promise but require further validation [3]. A more personalized and integrated approach, which incorporates both physical and psychological support, is essential to address the multifactorial nature of BPPV in this population [3]. Advancements in diagnostic technology, alongside a multidisciplinary care approach, will be crucial in improving patient outcomes and providing more effective, evidence-based treatment for TBI-related BPPV [3].

Challenges in Neuroimaging of Traumatic Brain Injury: Insights From MRI, DTI, and Vestibular Dysfunction

Neuroimaging plays a crucial role in understanding the pathophysiology of traumatic brain injury (TBI) [56]. However, despite advances in magnetic resonance imaging (MRI) and diffusion tensor imaging (DTI), significant challenges remain in accurately diagnosing and characterizing TBI, particularly in mild cases (mTBI) [57]. The heterogeneity of TBI, subtle structural changes, and limitations in current imaging techniques make it difficult to establish definitive biomarkers for prognosis and treatment response [57]. Furthermore, the interplay between TBI and vestibular dysfunction, including benign paroxysmal positional vertigo (BPPV), adds another layer of complexity to neuroimaging interpretation [4]. 

MRI Findings and Limitations in TBI

**Figure 2:**
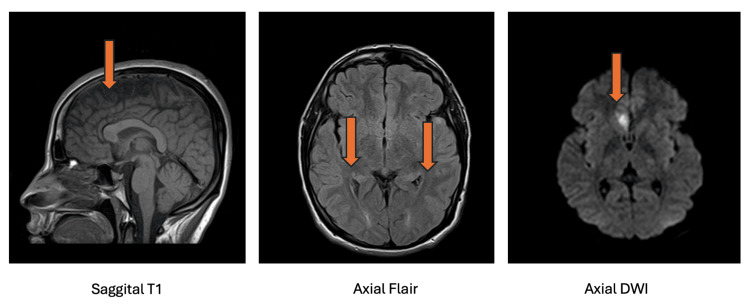
Areas of high FLAIR signal with restricted diffusion on axial DWI are observed in the corpus callosum (genu and splenium), the subcortical white matter of the right temporal lobe, the left occipital region, and the posterior parasagittal parietal regions, consistent with DAI grade II (arrows). FLAIR: Fluid-Attenuated Inversion Recovery; DWI: diffusion-weighted imaging; DAI: diffuse axonal injury The images are taken from Haouimi (2025) [58]. This content is covered under CC BY-NC-SA 3.0.

MRI is a widely used tool for detecting brain abnormalities in TBI patients [57]. It can reveal brain atrophy, microhemorrhages, and white matter abnormalities, particularly in moderate-to-severe cases [57]. One of the most consistent findings is overall brain volume reduction, including atrophy of gray and white matter structures [57]. Frontal and temporal lobe volume loss has been linked to cognitive deficits, while hippocampal atrophy is associated with memory impairment [57]. Additionally, MRI has identified corpus callosum damage, a key region affected in diffuse axonal injury (DAI). However, conventional MRI often fails to detect microstructural damage in mTBI, leading to diagnostic uncertainty [57]. Diffuse axonal injury (DAI), also known as traumatic axonal injury (TAI), is a severe form of traumatic brain injury caused by shearing forces. High Fluid-Attenuated Inversion Recovery (FLAIR) signal areas with restricted diffusion are observed in the corpus callosum (genu and splenium), the subcortical white matter of the right temporal lobe, the left occipital region, and the posterior parasagittal parietal regions, consistent with diffuse axonal injury grade II. As shown in Figure 2, these areas of restricted diffusion highlight the characteristic involvement of DAI.

Advances and Challenges in Diffusion Tensor Imaging (DTI)

DTI has emerged as a sensitive technique for detecting axonal injury in TBI, particularly in cases where conventional MRI and CT appear normal [57]. By measuring fractional anisotropy (FA) and mean diffusivity (MD), DTI provides insights into microstructural changes in white matter tracts [59]. Studies have reported reduced FA in the corpus callosum, internal capsule, and centrum semiovale in mTBI, suggesting compromised axonal integrity [60]. Additionally, increased MD in the splenium of the corpus callosum has been observed in both acute and chronic phases of injury, highlighting the evolving nature of TBI pathology [60]. Despite these advantages, the interpretation of DTI findings remains challenging due to variability in acquisition parameters, lack of standardized reference atlases, and the influence of confounding factors such as age and pre-existing conditions [61].

Whole-Brain Voxel-Wise DTI Analysis in TBI

Recent whole-brain voxel-wise DTI studies have aimed to address regional variations in white matter damage across different TBI severities [62]. These studies have demonstrated diffuse alterations beyond the classic areas of DAI, with reductions in FA observed in subcortical structures, thalamus, and cingulate gyrus [62]. Voxel-wise approaches provide a more comprehensive assessment of injury patterns but require advanced computational methods and large normative datasets for accurate interpretation [62]. As machine learning and artificial intelligence techniques advance, voxel-wise DTI may become a more reliable tool for individualized TBI assessment [62].

The Link Between TBI and Vestibular Dysfunction: Implications for Neuroimaging

A significant subset of TBI patients experiences vestibular dysfunction, with BPPV being one of the most common post-traumatic sequelae [4]. BPPV following TBI is thought to result from otoconial dislodgment due to head trauma, affecting the semicircular canals and leading to positional vertigo [16]. While neuroimaging techniques such as MRI and DTI have not been specifically designed to detect vestibular disorders, emerging research suggests that structural and functional changes in the brainstem, cerebellum, and vestibular pathways may contribute to post-traumatic dizziness [16]. Functional MRI (fMRI) and high-resolution diffusion imaging hold promise for investigating the neural correlates of vestibular impairment in TBI patients, potentially guiding more targeted rehabilitation strategies [63].

**Figure 3:**
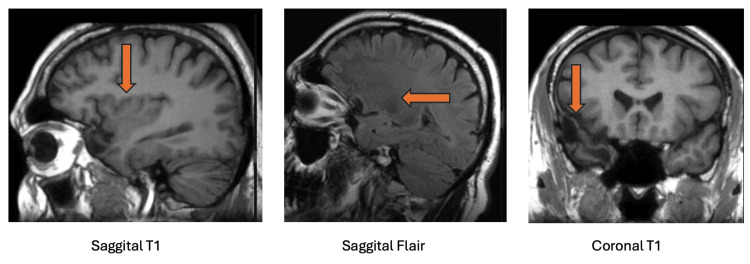
Extensive encephalomalacia and gliosis are observed, predominantly in the temporal pole and, to a lesser degree, on the inferior surface of the frontal lobes. This pattern is characteristic of a prior closed head injury (arrows). The images are taken from Schubert (2011) [65]. This content is covered under CC BY-NC-SA 3.0.

A subset of patients diagnosed with benign paroxysmal positional vertigo (BPPV) report symptom onset following head trauma, highlighting trauma as a potential trigger for BPPV [64]. Although only a small percentage of cases identify a traumatic event as the initiating factor, magnetic resonance imaging (MRI) findings provide valuable insights into this association [64]. Notably, patients with chronic dizziness are more likely to have a history of cerebral contusion or old hemorrhage [64]. These lesions, likely resulting from previous trauma, suggest that structural brain changes may contribute to persistent vestibular symptoms [64]. Additionally, brain atrophy, observed in a significant portion of patients, has been identified as an independent predictor of prolonged dizziness following BPPV [64]. While traumatic BPPV cases are relatively rare, MRI findings such as old contusions, atrophy, areas of encephalomalacia, and gliosis underscore the importance of neuroimaging in selected patients, particularly those with atypical presentations, prolonged symptoms, or a clear history of head trauma [64]. These findings support a more comprehensive diagnostic approach that incorporates MRI when trauma-related BPPV is suspected [63]. Multiple areas of encephalomalacia and gliosis, most prominent in the temporal pole and, to a lesser extent, on the inferior surface of the frontal lobes. This pattern of encephalomalacia and gliosis is characteristic of a prior closed head injury (Figure 3).

Traumatic injuries represent a significant etiological factor in benign paroxysmal positional vertigo (BPPV), particularly involving the posterior semicircular canal (pSCC) [66]. In a large retrospective study involving 500 patients with pSCC BPPV, trauma was identified as the most common identifiable cause, accounting for 16% of cases [66]. Mechanical trauma to the head and neck was especially prevalent among younger patients, highlighting an age-related pattern in BPPV onset [66]. Notably, brain MRI findings in these patients revealed that intracranial hemorrhages, particularly microhemorrhages and contusions, were more frequently observed in younger individuals with a traumatic history [66]. These findings underscore the importance of considering MRI as a diagnostic adjunct in BPPV cases with a traumatic onset, not only to explore underlying central pathologies but also to guide appropriate management and referrals [66]. The association between trauma-induced BPPV and abnormal MRI findings reinforces the need for heightened clinical vigilance in such presentations [66].

## Conclusions

BPPV secondary to TBI represents a complex interplay of pathophysiological mechanisms, diagnostic challenges, and management dilemmas. While significant progress has been made in understanding this condition, many questions remain unanswered, particularly regarding long-term outcomes and the optimization of therapeutic strategies. Clinicians must adopt a holistic, patient-centered approach that considers the unique needs of TBI survivors, integrating advances in diagnostic technologies and rehabilitation modalities. Future research should focus on elucidating the underlying mechanisms of post-traumatic BPPV, identifying predictive factors for recurrence, and developing evidence-based guidelines for its management. Such efforts are critical to improving the quality of care and outcomes for this vulnerable patient population.
